# Pericytes: brain-immune interface modulators

**DOI:** 10.3389/fnint.2013.00080

**Published:** 2014-01-10

**Authors:** Gabriela Hurtado-Alvarado, Adrian M. Cabañas-Morales, Beatriz Gómez-Gónzalez

**Affiliations:** Area of Neurosciences, Department of Biology of Reproduction, Unidad Iztapalapa, Universidad Autónoma MetropolitanaMexico City, Mexico

**Keywords:** pericytes, blood–brain barrier, immune response, inflammation, cytokines, REM sleep loss, brain endothelial cell, tight junction disruption

## Abstract

The premise that the central nervous system is immune-privileged arose from the fact that direct contact between immune and nervous cells is hindered by the blood–brain barrier. However, the blood–brain barrier also comprises the interface between the immune and nervous systems by secreting chemo-attractant molecules and by modulating immune cell entry into the brain. The majority of published studies on the blood–brain barrier focus on endothelial cells (ECs), which are a critical component, but not the only one; other cellular components include astroglia, microglia, and pericytes. Pericytes are poorly studied in comparison with astrocytes or ECs; they are mesenchymal cells that can modify their ultrastructure and gene expression in response to changes in the central nervous system microenvironment. Pericytes have a unique synergistic relationship with brain ECs in the regulation of capillary permeability through secretion of cytokines, chemokines, nitric oxide, matrix metalloproteinases, and by means of capillary contraction. Those pericyte manifestations are related to changes in blood–brain barrier permeability by an increase in endocytosis-mediated transport and by tight junction disruption. In addition, recent reports demonstrate that pericytes control the migration of leukocytes in response to inflammatory mediators by up-regulating the expression of adhesion molecules and releasing chemo-attractants; however, under physiological conditions they appear to be immune-suppressors. Better understanding of the immune properties of pericytes and their participation in the effects of brain infections, neurodegenerative diseases, and sleep loss will be achieved by analyzing pericyte ultrastructure, capillary coverage, and protein expression. That knowledge may provide a mechanism by which pericytes participate in the maintenance of the proper function of the brain-immune interface.

## INTRODUCTION

The brain must respond to blood-borne signals but has no direct access to them (Persidsky et al., 2006; Saper, 2010). Likewise, the immune system does not contact directly the brain *milieu*; they interact through the brain-immune interface, the blood–brain barrier. The interface is comprised by endothelial cells (ECs), astrocytes, microglia, pericytes, and extracellular matrix components (basal lamina and glycocalyx; Risau, 1991; Ballabh et al., 2004; Ueno, 2007; Gómez-González et al., 2012). ECs limit blood-borne macromolecules or cells from crossing into the brain through junction complexes that fasten together adjacent cell membranes. In addition, transcellular trafficking of molecules is limited by the minimal expression of endocytosis and the presence of specialized carrier systems (Zlokovic, 2008; Abbott et al., 2010). Although ECs provide the physical and chemical barrier function *per se*, all elements are crucial for the development and maintenance of the blood–brain barrier, allowing it to be the interface between peripheral systems and the brain (Zlokovic, 2008).

Pericytes have been increasingly implicated in the regulation of local blood-flow in brain regions with increased synaptic activity, a phenomenon known as neurovascular coupling (reviewed in Hamilton et al., 2010); furthermore, they have also been involved in the regulation of the blood–brain barrier permeability to circulating molecules (Armulik et al., 2010). Better understanding of the immune properties of pericytes and their participation in the changes observed during brain infections and neurodegenerative diseases will provide a mechanism by which pericytes participate in the maintenance of the proper function of the brain-immune interface, the blood–brain barrier. Here we present recent evidence depicting the new roles of pericytes in regulating blood–brain barrier function under normal and pathological conditions and hypothesize its potential role in the regulation of the blood–brain barrier after chronic sleep loss.

### PERICYTES AS BLOOD–BRAIN BARRIER COMPONENTS

Pericytes are smooth muscle-derived cells that play a crucial role in keeping brain homeostasis given their presence at the blood–brain barrier and particularly their active role in what is known as the neurovascular unit (Zlokovic, 2008; Gómez-González et al., 2012). Rouget (1874), for the first time, described a population of branched cells with contractile properties that surrounded ECs. Fifty years later, these mesenchymal cells were renamed “pericytes” by Zimmerman in concordance with their anatomical location: abluminal to ECs and luminal to parenchymal cells (Kim et al., 2006; Sá-Pereira et al., 2012). Anatomically, pericytes have projections that wrap around capillaries and are embedded within the basal lamina. The diversity in pericyte marker expression may be related to vessel size or embryonic origin; the main markers are α-smooth muscle actin (αSMA), desmin, the regulator of G-protein signaling 5 (RGS-5), neuron-glial antigen 2 (NG2), platelet-derived growth factor receptor (PDGFRα and PDGFRβ), and amino-peptidase-N (CD13; Ozerdem et al., 2002; Bergers and Song, 2005). These proteins show different expression patterns under physiological and pathological states (see **Table 1**). Furthermore, pericytes express numerous macrophage markers, namely CD4, CD11b, CD146, and proteins related to immune function such as the fragment crystallizable receptor (FcR) and the major histocompatibility complex (MHC) classes I and II (Bergers and Song, 2005; Kamouchi et al., 2011). Differences in the expression of those markers are based on the local environmental influences on pericytes. For example, it has been reported that CD146 is expressed during embryonic development but not in all freshly isolated pericytes in adulthood. Also, RGS-5 protein expresses during embryonic development, but decreases after birth and is absent in pericytes of the normal adult central nervous system (Dore-Duffy, 2008; Sá-Pereira et al., 2012).

**Table 1:** Pericyte markers in health and disease.

**Pericyte marker/location**	**Main function**	**Main physiological role**	**Health**	**Disease**	**Reference**
**PDGFRβ**/cell surface protein	Tyrosine-protein kinase; Kinase receptor	Embryonic development, proliferation, chemotaxis, host-virus interaction	+	+/- Fibrosis Tumor Blood–brain barrier disruption	Song et al. (2005), Armulik et al. (2010), Dore-Duffy and Cleary (2011)
**αSMA**/Filament protein	Contractility	Regulation of blood flow and motility	-	++ Fibrosis Tumor Blood–brain barrier disruption	Song et al. (2005), Dore-Duffy and Cleary (2011)
**NG2**/cell surface protein	Cell adhesion protein	Vasculo-genesis	+	+Fibrosis Tumor Blood–brain barrier disruption	Ozerdem et al. (2002), Dore-Duffy and Cleary (2011)
**RGS**-5/intracellular protein	GTPase-activating protein	Cell motility	+	++ Fibrosis Tumor Blood–brain barrier disruption	Song et al. (2005), Dore-Duffy and Cleary (2011)
**Desmin**/filament protein	Contractility	Regulation of blood flow and motility	+	+ Fibrosis Tumor Blood–brain barrier disruption	Dore-Duffy and Cleary (2011), Kamouchi et al. (2011)
**CD13**/cell surface protein	Ectopeptidase	Pericyte differentiation	+	++ Fibrosis Tumor Blood–brain barrier disruption	Armulik et al. (2010), Kamouchi et al. (2011)

*Symbols are as follow: (+) Indicates that the marker is present; (-) indicates that the marker is absent; (+/-) indicates a decrease in marker expression and; (++) indicates that the marker is over expressed.*

**FIGURE 1:**
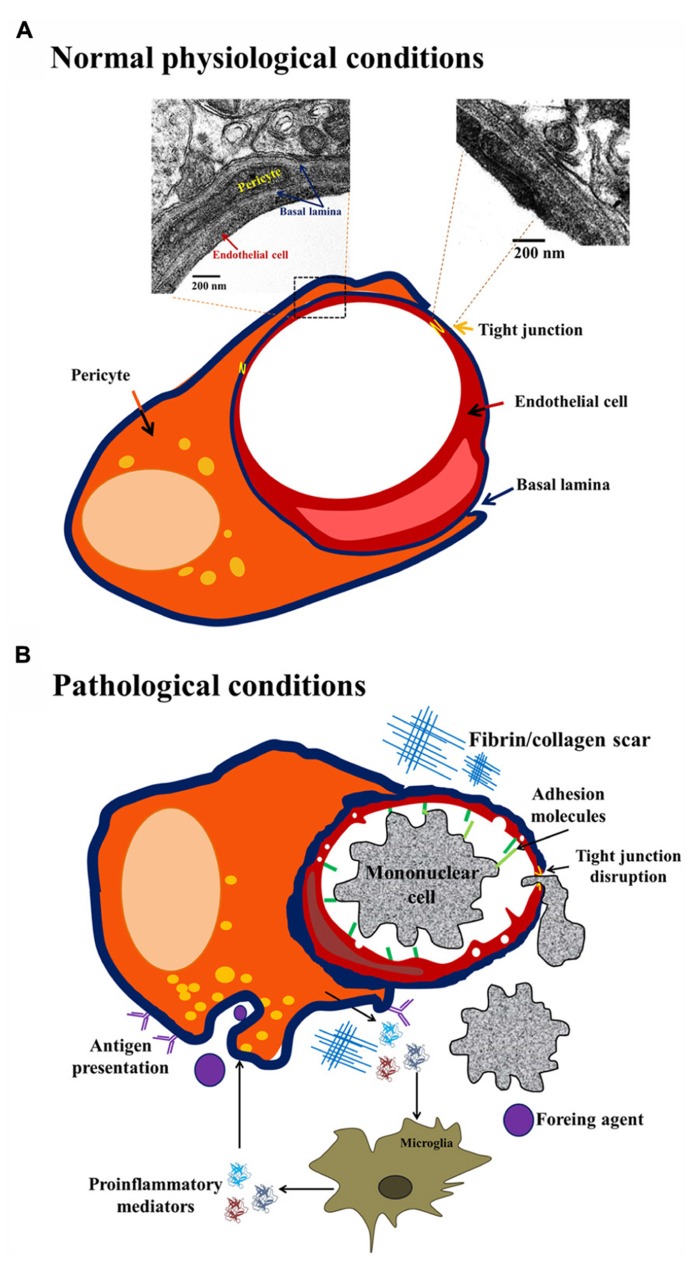
**Brain pericyte phenotype in normal and pathological conditions.** Under normal physiological conditions **(A)** brain pericytes exhibit tight junctions (Tjs) with endothelial cells (ECs), and are embedded in the basal lamina. Under pathological conditions, such as injury, infection or neurodegeneration **(B)**, pericytes present a migrating phenotype with up-regulation of ICAM expression, pro-inflammatory cytokine release with ensuing recruitment of peripheral mononuclear cells. Additionally, under pathological conditions, the continuity of basal lamina is lost and the existence of fibrin scars contributes to blood–brain barrier impairment.

Although pericyte identification is rather difficult owing to the lack of one specific marker (Özen et al., 2012), its ultrastructure was described (Nag, 2003; Sá-Pereira et al., 2012). Two classes of pericytes exist in the brain: granular and agranular; this classification arises from the presence or absence of lysosome-like granules in the cytoplasm (Farrell et al., 1987). In humans, less than 5% of the pericyte population is agranular (Farrell et al., 1987; Nag, 2003). Both, granular and agranular pericytes exhibit an oval cell body and a prominent round nucleus that is different from the elongated nucleus of ECs. Each pericyte may cover 100 μm of capillary length with up to 90 ramifications 300–800 nm wide (Nag, 2003; Sá-Pereira et al., 2012). Pericyte distribution is intermittent along the walls of arterioles, venules and, particularly, in capillaries (Dore-Duffy, 2003). They are crucial for the development and maintenance of the main nervous system barriers, namely, blood–spinal cord barrier, blood–retinal barrier, blood–nerve barrier and blood–brain barrier. In fact, pericyte coverage of brain ECs *in vitro* is approximately 80%, in the capillaries of the retina it is 90%, and in the microvessels of the spinal cord it is less than 60%. Pericyte coverage and number is related to the permeability of the biological-barriers, higher coverage correlates with lower permeability (Winkler et al., 2012). Specifically, it has been shown that pericytes contribute to regulate capillary structure and diameter (Peppiatt et al., 2006; Armulik et al., 2010; Bell et al., 2010; Daneman et al., 2010). Pericytes express junctional complexes that include gap junctions, tight junctions (Tjs), and focal adhesions with ECs (Zlokovic, 2008). These associations lead to the maintenance of low permeability of the cerebral endothelium (Lai and Kuo, 2005; Nakagawa et al., 2007). Brain pericytes promote a reduction in vesicular transport, (Daneman et al., 2010), and promote endothelial Tj protein expression (Zonula occludens, ZO-1, claudin-5, occludin; **Figure 1**; Armulik et al., 2005,^2010^; Daneman et al., 2010). In addition, the morphological pattern of pericyte projections around brain capillaries is linked to their function and intimately correlates with brain health state (normal, angiogenic, or injured; Dore-Duffy and Cleary, 2011). The classic wrapping pattern consists of broad processes with a large continuous surface in the external wall of brain microvessels (Dore-Duffy, 2003; Nag, 2003; Dore-Duffy and Cleary, 2011). Under normal conditions, the wrapping pattern predominates, but in pathological conditions detachment and migrating patterns can be observed with the formation of finger-like projections followed by retraction of projections (**Figure 1**; Dore-Duffy and Cleary, 2011). Different morphological patterns in pericyte processes may appear in response to changes in the microenvironment. For example, the migrating pattern is associated to up-regulation of cell surface proteases in aversive conditions, and also with early stages of angiogenesis, in contrast with the wrapping pattern that predominates in normal capillaries (Dore-Duffy, 2003; Sá-Pereira et al., 2012).

Morphological changes in pericytes vary as a function of exposure to soluble molecules released by blood–brain barrier components such as ECs, neurons, microglia or astrocytes; pericytes can differentiate into fibroblasts, smooth muscle cells or macrophages, depending on the stimulus received (**Figure 1**). The molecules released to the basal lamina that can promote pericyte morphological changes include neurotransmitters, neuro-hormones and inflammatory mediators (Özen et al., 2012). To illustrate this, it has been shown that adenosine and adenosine triphosphate (ATP) released by neurons and glial cells may modify pericyte status by activating purinergic receptors; in addition, rat brain pericytes express ecto-nucleotidase 1 and 2 (Ceruti et al., 2011; Lecca et al., 2012). After immune challenges such as lipopolysaccharide (LPS) administration, hippocampal brain pericytes present increased ecto-nucleotidase expression and function and also morphological changes (Kittel et al., 2007). Activation of purinergic receptor P2X7 initiates an inflammatory response by inducing interleukin (IL) 1β secretion from ECs, astrocytes, microglia, and also pericytes (Derks and Beaman, 2004; Lecca et al., 2012).

Pericyte versatility is, for the most part, unexplored, but several studies suggest that pericytes may play potential roles in brain repair through contractile, migratory, pro-angiogenic and phagocytic functions but they can also promote brain impairment by uncontrolled immune response (Dore-Duffy et al., 2000; Dore-Duffy et al., 2006; Özen et al., 2012; Sá-Pereira et al., 2012).

### IMMUNE PROPERTIES OF BRAIN PERICYTES

Mesodermal or neural crest origins of pericytes are generally accepted. Pericytes are considered as “brain macrophages”. In fact, for some authors, they represent the first line of defense in the central nervous system due to their antigen presentation properties and because they are directly associated with the microvasculature, in contrast to microglia (**Figure 1**; Balabanov et al., 1999; Guillemin and Brew, 2004). Thomas (1999) reported pericytes leaving the basal lamina and migrating to the perivascular space where they are indistinguishable from perivascular macrophages and reactive microglia (Guillemin and Brew, 2004). Pericyte de-differentiation into cells presenting antigens may initiate a local pro-inflammatory response. Immune response in the brain induces monocyte and lymphocyte recruitment; this process is mediated by the increased expression of adhesion molecules (e.g., intracellular adhesion molecule 1, ICAM-1) in the luminal region of ECs that correlates with decreases in the number of Tjs (**Figure 1**; Guillemin and Brew, 2004). In addition, pericytes are able to produce chemo-attractants and promote transmigration to the brain of circulating immune cells, starting an inflammatory process. Pericytes may also release inflammatory mediators, such as IL-1β, IL-6, tumor necrosis factor (TNF) α, reactive oxygen species, nitric oxide (NO), and matrix metalloproteinases (MMP-2 and MMP-9), all of which contribute to pericyte detachment and blood–brain barrier disruption (Kovac et al., 2011).

These immunoactive properties of pericytes suggest mechanisms by which they can act as an integral part of the blood–brain barrier during brain inflammatory processes. A pro-inflammatory component is the hallmark of several brain diseases. Vascular damage associated to pericyte deficiency may precede neurodegeneration in brain infections, Alzheimer’s or Parkinson’s disease, diabetes (Özen et al., 2012), and perhaps in less-explored phenomena that exhibit considerable cognitive impairments, such as sleep loss.

### PERICYTES AND BRAIN INFECTIONS

The blood–brain barrier provides a shield against foreign agents that initiate inflammatory responses (Al-Ghananeem et al., 2013). The structural variability and the nature of biotic/abiotic inflammatory agents that may promote neuropathology are reflected in the mechanisms used to access the brain. These mechanisms include receptor-mediated endocytosis, unspecific transport by pinocytotic vesicles, paracellular diffusion, transmigration through infected leukocytes, and crossing after blood–brain barrier breakdown (Alcendor et al., 2012; Nakagawa et al., 2012; Pulzova et al., 2012).

The inflammatory response to a foreign agent may cause irreversible brain damage by continuous exposure to pathogen-derived toxic molecules and immune mediators (Kumar et al., 2009; Hirooka and Kaji, 2012). Factors that promote a pro-inflammatory state in the brain include abiotic agents such as heavy metal ions or viruses, and biotic factors such as bacteria, fungi, and parasites (Gasque et al., 1998; Liou and Hsu, 1998; Alvarez and Teale, 2007; Hirooka and Kaji, 2012; Nakagawa et al., 2012). There is scant knowledge of pericyte function and structure under inflammatory response induced by foreign agents.

Heavy metal ions, such as methyl-mercury, cadmium and inorganic mercury induce a potent inflammatory response in the brain. These metal ions have high affinity to sulfhydryl groups favoring the formation of a methionine-like complex that easily crosses the blood–brain barrier. The methionine-like complex enters the brain by the large neutral amino acid transporter (LAT-1); once inside, the heavy metal ions induce cytokine and growth factor release by blood–brain barrier components. Heavy metal ions associate with the fibroblast growth factor type 2 (FGF-2); this union may cause cell damage because FGF-2 is unable to repair endothelial damage; therefore, heavy metal ions promote less auto-regulatory signaling inhibition of EC proliferation (Hirooka and Kaji, 2012).

In the case of viral and bacterial infections, such as congenital human cytomegalovirus (HCMV), human immunodeficiency virus type 1 (HIV-1), Japanese encephalitis (JE) virus and bacterial meningitis, the main transport routes through the blood–brain barrier include endocytosis of blood-circulating vesicles, microvessel wall degradation, and indirect crossing via previous blood–brain barrier disruption. When infectious agents are detected, pericytes begin an inflammatory response through increased expression of pro-inflammatory cytokines, such as IL-1β, IL-6, and TNF-α (Liou and Hsu, 1998; Alcendor et al., 2012; Nakagawa et al., 2012). In HIV-1 infection, pericytes express the chemokine receptors CXCR4 and CCR5 that are used by infected cells to contribute to the formation of viral reservoirs in the brain (Nakagawa et al., 2012). It is known that 80% of cultured pericytes infected by HCMV generate an inflammatory response; in fact, only 72 h after infection, a huge rise in IL-1β, a medium increase in IL-6, and a minimal increase in TNF-α concentration are observed. However, later on those pro-inflammatory cytokine profiles are reversed by the compensatory effect of anti-inflammatory cytokines (Alcendor et al., 2012). In contrast, bacterial meningitis infection increases expression of receptors C5a and C3a in brain pericytes. These complement molecules are powerful chemo-attractants to recruit polimorfonuclear cells and macrophages to the inflammation site causing cell activation (Gasque et al., 1998). On the other hand, it has been reported that *Taenia solium* infiltrates cause brain inflammation by pericyte release of pro-inflammatory cytokines and MMP-2 and MMP-9, which are associated to blood–brain barrier disruption. Blood–brain barrier breakdown allows infiltration of antigen-presenting cells and specialized immune cells (B cells and T cells), exacerbating the inflammatory condition (Alvarez and Teale, 2007).

**FIGURE 2:**
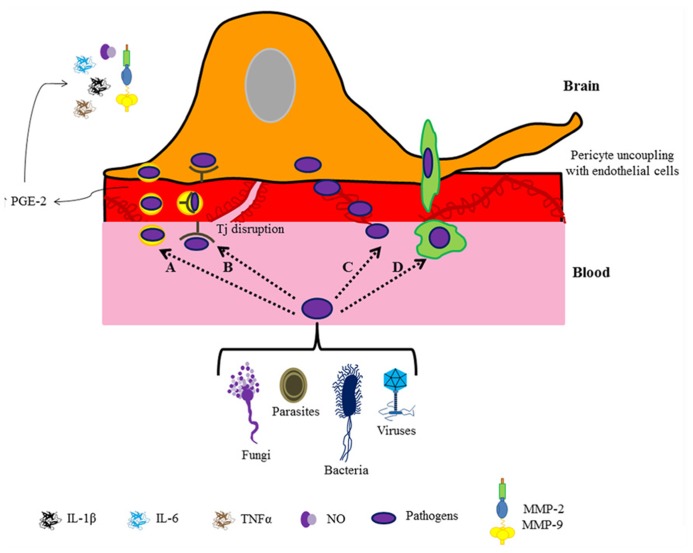
**Pathogen entry to the brain.** The cartoon depicts different routes for pathogen entry to the brain: caveolae **(A)**, receptor-mediated transport **(B)**, paracellular diffusion **(C)**, and transmigration through infected leukocytes **(D)**. In all cases a pericyte-mediated immune response followed by blood–brain barrier breakdown is observed.

These studies illustrate that although each pathogen exhibits a characteristic pathway, the same inflammatory mediators participate in the orchestration of the brain immune response (**Figure 2**). It is known that rises in pro-inflammatory cytokines, particularly IL-1 β, IL-6, and TNF-α, disrupt Tjs by down-regulating occludin and ZO-1 expression (Liou and Hsu, 1998; Alcendor et al., 2012; Nakagawa et al., 2012). Pro-inflammatory cytokines alter Tj integrity by promoting an increase in prostaglandin-E (PGE) receptors in pericytes, which leads to MMP overproduction and release, causing pericyte uncoupling with ECs (Alvarez and Teale, 2007). In fact, ECs are the unique brain cell type that expresses PGE-2 synthase (Yamagata et al., 2001); PGE-2 is produced in response to immune challenges (e.g., IL-1 or LPS administration; Cao et al., 1997; Laflamme et al., 1999) suggesting a relevant role of perivascular cells (astrocytes, interneurons and particularly pericytes) in the response to low doses of immune stimulators (Schiltz and Sawchenko, 2002). Interestingly, perivascular cell response is different for each type of molecule; e.g., pericytes elicit cyclooxygenases in brain ECs in response to low doses of IL-1, but with low doses of LPS perivascular cells apparently have an inhibitory effect on cyclooxygenase production (Schiltz and Sawchenko, 2002). Some neuro-infections are associated with neurodegenerative diseases, for example, the bacteria *Borrelia burgdorferi* in Alzheimer’s disease (Miklossy et al., 2004). So, is the pathogenic action on pericytes a promoter of neurodegenerative disease? Undoubtedly, pericyte function has an important role in the progression of brain pathologies. Although several studies provide relevant information on the immune role of pericytes in the protection of the brain against an infectious threat, the molecular and cellular mechanisms involved in blood–brain barrier disruption are poorly understood.

### ROLE OF PERICYTES IN NEURODEGENERATIVE DISORDERS

**FIGURE 3:**
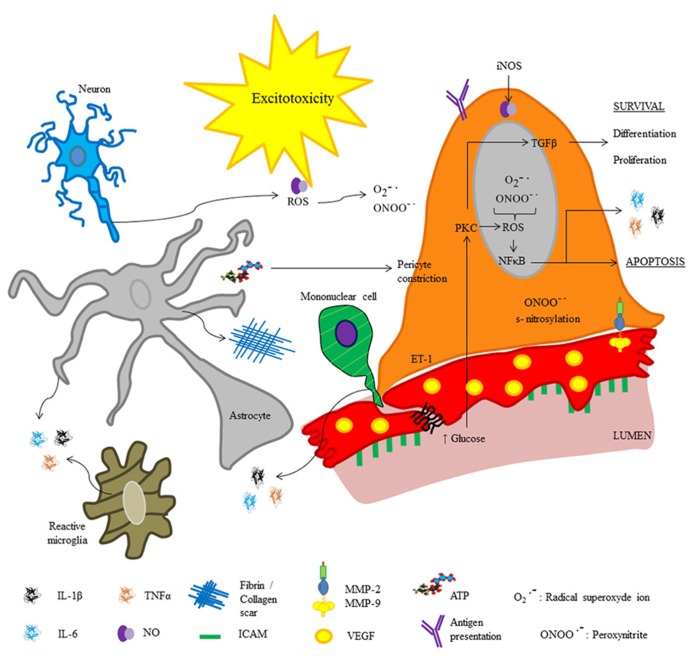
**Molecular inflammatory changes in brain pericytes in pathophysiological conditions.** The cartoon depicts molecules released by pericytes under altered physiological conditions (e.g., neurodegeneration, infections or brain injury). Excitotoxicity may occur secondary to blood–brain barrier disruption.

Similar to infectious processes, during neurodegenerative and cerebrovascular diseases inflammatory phenomena occur, which are characterized by increased release of pro-inflammatory cytokines (IL-1β, IL-6, and TNF-α), subsequent hyperthermia, and mononuclear cell infiltration (Bleys and Cowen, 2001). In both, neurodegenerative and cerebrovascular diseases, pericyte detachment of ECs and differentiation into fibroblasts or phagocytes correlates with an increase in vesicle number in ECs, Tj disruption and immune cell recruitment (Özen et al., 2012). Additionally, fibrosis-like pathophysiological changes are described (**Figure 3**); pericyte-derived fibrin and collagen form scars, which are involved in cell death by neurotoxicity (Armulik et al., 2010; Fernández et al., 2013). Deposits of extracellular matrix components and organ failure are common after prolonged exposure to pro-inflammatory cytokines, suggesting that the first step leading to cell death relates to the immune response (Lin et al., 2008; Armulik et al., 2010). Furthermore, cytokine production is accompanied by oxidative stress; both, inflammatory mediators and oxidative stress are directly involved in increased blood–brain barrier permeability through the same signaling pathways.

Recent studies revealed the role of NO released by microglia and pericytes in neurodegenerative diseases and neuro-immune interactions; it was shown that amyloid β deposits in Alzheimer’s disease promote pericyte constriction despite NO over-production. The role of NO in blood–brain barrier disruption is also related to its high ability to form free radicals such as peroxynitrite (ONOO-•), which may induce cell death (Hamilton et al., 2010; Kovac et al., 2011). In addition, it has been reported that amyloid β deposits promote over-production of reactive oxygen species in pericytes, endothelial, and glial cells (Veszelka et al., 2013). Blood–brain barrier disruption promotes lymphocyte recruitment in neurodegenerative diseases and stroke; hence, after cerebral ischemia, polymorphonuclear leukocytes impede reperfusion leading to generation of free radicals, and promoting pericyte constriction. Indeed, pericyte detachment from the vessel wall occurs following ischemia and reperfusion (Takahashi et al., 1997). Recently, Tigges et al. (2013) reported an increase in fibronectin and collagen I deposits in animal models of Alzheimer’s disease, these deposits are related to pericyte differentiation and migration. Tigges et al. (2013) showed that under normal conditions, brain pericytes express high levels of α5 integrin and lower levels of α1, α2, and α6 integrins. This expression pattern has a crucial role in the attachment of pericytes to the vessel wall; in fact, an *in vivo* study shows that TNF-α promotes pericyte proliferation and detachment as well as a switch in integrin expression pattern, with predominance of α2 integrin (Tigges et al., 2013). Interestingly, Tigges et al. (2013) also found that α2 integrin expression strongly correlated with brain vessel remodeling in experimental autoimmune encephalomyelitis. Similarly, in Alzheimer’s disease it is reported that fibrin deposition and increased extravascular immunoglobulin G (IgG) correlate with a reduction in pericyte coverage of ECs (Sengillo et al., 2013).

Fibrin deposits are a signal of fibroblast activity and probably represent an index of de-differentiation from pericytes to fibroblasts. Transforming growth factor-β (TGF-β) is the most potent known growth inhibitor for ECs, fibroblasts, neurons, and lymphoid cells. TGF-β inhibits proliferation of T-lymphocytes by down-regulating pro-inflammatory cytokines, e.g., IL-2-mediated proliferative signals (Dohgu et al., 2005). Under diabetic conditions, pericytes release TGF-β, which increases fibronectin levels (Shimizu et al., 2013). Shimizu et al. (2013) suggest that advanced glycation end-products (AGEs) induce blood–brain barrier disruption in diabetic conditions by stimulation of autocrine TGF-β signaling in pericytes, and up-regulation of vascular endothelial growth factor (VEGF) and MMP-2. Both, VEGF and MMP-2 modify trans-endothelial electric resistance (TEER) leading to Tj disruption and increased vesicular transport (Thanaóbalasundaram et al., 2011). Pericyte deficiency reported in diabetes is attributed to raises in glucose concentration, and production of reactive oxygen species through the NFκB pathway (Hamilton et al., 2010). Interestingly, in diabetic animal models, pericytes are highly immunosuppressive; under early hyperglycemic conditions retinal-derived pericytes inhibit T cell proliferation and protect ECs from inflammation-induced apoptosis (Tu et al., 2011). In addition, it is known that pericytes are especially susceptible to oxidative stress; for example, high glucose levels cause oxidative stress and apoptosis (Shah et al., 2013). In addition to the reactive oxygen species effect, the production of large amounts of NO by inducible-nitric oxide synthase (iNOS) can lead to changes in cerebral blood-flow, nitrosative stress, and subsequent cell death of pericytes, ECs and neurons through toxicity caused by excitatory amino acids and massive entry of toxic molecules to the brain (Kischer, 1992; Li et al., 1997; Tu et al., 2011; Baloyannis and Baloyannis, 2012). A decrease in pericyte capillary coverage and cell number has been reported in hyperglycemia, early diabetes retinopathy, brain tumors, and Alzheimer’s disease. Therefore, brain microvascular alterations seem to reciprocally interact with underlying neurodegeneration in inducing cognitive impairments (Pimentel-Coelho and Rivest, 2012). The role of pericytes in the genesis of neurodegenerative diseases and in brain regeneration is poorly studied; however, pericytes undoubtedly, cause alterations in brain physiology.

### PERICYTES AND SLEEP LOSS: AN IMMUNOLOGICAL PERSPECTIVE

Sleep loss is a common problem in modern society (Mills et al., 2007; Yehuda et al., 2009) and a risk factor for the development of obesity, metabolic syndrome, diabetes, and neurodegenerative diseases (Tasali et al., 2009; van Leeuwen et al., 2009; Reynolds et al., 2012). Similar to infections and neurodegenerative diseases, sleep loss has an important pro-inflammatory component (Mills et al., 2007; Zager et al., 2007). Specific sleep function is yet unclear; but it has been proposed that sleep is associated with changes in parameters of host defense (Benca and Quintas, 1997). Sleep is divided into two distinct stages namely; slow wave sleep and rapid eye movement (REM) sleep (Siegel, 2010). Particularly, REM sleep has an important role in biological processes; REM sleep loss decreases neurogenesis in the hippocampus (Guzman-Marin et al., 2008; Mueller et al., 2008), alters the brain neurochemical content (Mohammed et al., 2011), and impairs learning and memory in both rodents and humans (Meerlo et al., 2008). Prolonged wakefulness promotes an increase of inflammatory mediators such as adenosine and NO (Kalinchuk et al., 2011; Cespuglio et al., 2012; Raymond et al., 2012), and increases plasma levels of IL-1β, IL-6, IL-17A, TNF-α (Yehuda et al., 2009), and endothelin-1 (ET-1; Mills et al., 2007). These changes may act directly on the blood–brain barrier components; for example, IL-1, IL-17, and ET-1 disrupt the blood–brain barrier (Banks et al., 1995; Blamire et al., 2000; Didier et al., 2003; Huppert et al., 2010). REM sleep deprivation also increases body temperature (Jaiswal and Mallick, 2009), which also disrupts the blood–brain barrier (Kiyatkin and Sharma, 2009).

**FIGURE 4:**
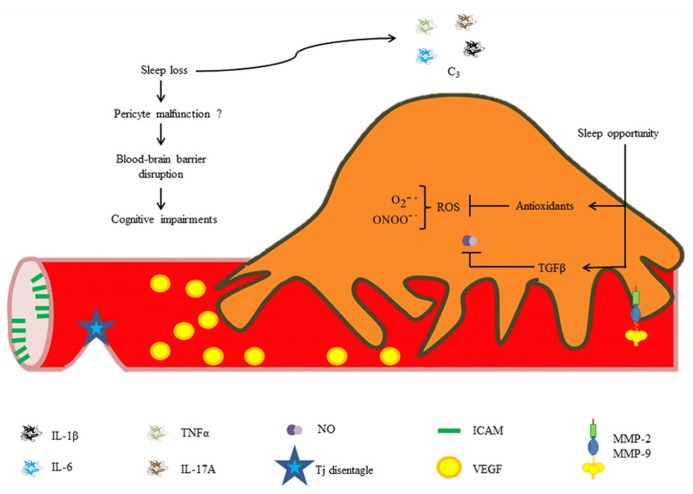
**Hypothetical roles of pericytes in REM sleep loss-induced blood–brain barrier disruption.** Increased cytokine and chemokine concentrations during sleep loss may disrupt the blood–brain barrier. Brief periods of sleep opportunity may reestablish the blood–brain barrier integrity through synthesis of antioxidants and anti-inflammatory cytokines (e.g., TGF-β).

Our research group recently found that chronic REM sleep restriction induces a generalized blood–brain barrier breakdown, and subsequent sleep opportunity is capable of restoring blood–brain barrier integrity. In addition, we studied EC ultrastructure and observed alterations in vesicle trafficking (Gómez-González et al., 2013). It is highly likely that pericyte dysfunction may contribute to increases in blood–brain barrier permeability secondary to sleep loss because ultrastructural changes in ECs are similar to those reported in pericyte-deficient mice, e.g., increased caveolae density, and endothelial derangement (Armulik et al., 2010). Chronic exposure to pro-inflammatory cytokines, NO and other inflammatory mediators released during sleep restriction may directly induce pericyte detachment from the vessel wall and subsequent differentiation into migratory and phagocytic phenotypes, mediating blood–brain barrier disruption. It is likely that the synthesis of antioxidants and anti-inflammatory molecules during sleep recovery may restore normal blood–brain barrier permeability through neutralization of free radicals (**Figure 4**).

## CONCLUSION

Classically, pericytes have been considered a cell population involved mainly in microvessel contractility. New research on pericyte contribution to optimal blood–brain barrier function and neural pathogenesis shows that they have a substantial influence on the neuro-immune response. The immunoactive properties of pericytes suggest mechanisms by which they could act as an integral component of the blood–brain barrier during inflammatory processes, such as during brain infections, neurodegenerative diseases or sleep loss. Future studies are needed to elucidate pericyte role under inflammatory conditions. Knowledge on pericyte contribution to disease pathogenesis will allow more specific treatment of brain pathologies and perhaps the development of better diagnostic markers. The field study of pericytes is generating frontier knowledge and may be exploited as an example of neuro-integration. Certainly, pericytes are crucial cells in optimal brain function, but their deficit results from molecular interactions between all brain cells.

## Conflict of Interest Statement

The authors declare that the research was conducted in the absence of any commercial or financial relationships that could be construed as a potential conflict of interest.
